# Predatory Medical Journals in Patent Literature: A Hidden Threat

**DOI:** 10.34172/apb.025.46153

**Published:** 2025-10-11

**Authors:** Mihály Hegedűs, Mehdi Dadkhah, Lóránt Dénes Dávid

**Affiliations:** ^1^Department of Finance and Accounting, Tomori Pál College, Budapest, Hungary; ^2^Chamber of Hungarian Auditors, Budapest, Hungary; ^3^Department of Sustainable Tourism, Institute of Rural Development and Sustainable Economy, Hungarian University of Agriculture and Life Sciences (MATE), Gödöllő, Hungary; ^4^Department of Tourism and Hospitality, Faculty of Economics and Business, John von Neumann University, Kecskemét, Hungary; ^5^Department of Tourism and Hospitality, Institute of Rural Development and Sustainable Economy, Hungarian University of Agriculture and Life Sciences (MATE), Gödöllő, Hungary; ^6^Savaria Department of Business Economics, Savaria University Centre, Faculty of Social Sciences, Eötvös Loránd University, Szombathely, Hungary; ^7^Department of Tourism and Hospitality, Kautz Gyula Faculty of Business and Economics, Széchenyi István University, HU9026 Győr, Hungary

**Keywords:** Predatory journals, Intellectual property, Patent, Circular economy, Sustainable development goals, Potential predatory journals

## Abstract

**Purpose:**

The negative impact of potential predatory journals has been widely discussed, primarily within academic contexts. However, their influence beyond academia remains underexplored. This study aims to address that gap.

**Methods:**

The current editorial utilised a sample list of 8 potential predatory medical journals. We compiled a list of potential predatory medical journals using the discontinued titles list in Scopus and the current blocklists. Then their patent-to-paper citations have been examined to understand the dissemination of questionable medical publications outside of academia.

**Results:**

This indicates that potential predatory medical journals received 483,848 citations from scholarly works and 4,251 citations from patents.

**Conclusion:**

When patents cite papers from predatory journals, flawed information may propagate, or potentially leading to wrongful patent rejections and wasted resources. This serves as a warning for the patent community to take action against potential predatory journals.

## Introduction

 Currently, academia is faced with the issue of potential predatory journals. There are numerous studies in this area, most of which focus on what is known as predatory journals.^1-4^ The term’ predatory journals’ refers to those that publish authors’ manuscripts without considering fair academic standards and usually by charging authors without providing a high-quality peer review.^5-7^ This term was coined by Jeffrey Beall in 2010 to describe the practice of these predatory publications; however, there is no consensus on a definition of them.^1,5^ The identification of predatory journals primarily relies on blocklists (e.g., Cabells) or criteria established by Jeffrey Beall, which have been revised or reformulated by other sources.^8-11^ Predatory journals yield numerous adverse effects, such as undermining the reputations of researchers and institutions, restricting research visibility, disseminating non-peer-reviewed or pseudo-scientific literature, squandering researchers’ time and institutional budgets, compromising academic integrity, exacerbating unethical publishing practices, jeopardizing clinical practice and patient safety, and adversely affecting sustainable development goals.^10,12-14^

 As published papers in predatory journals often suffer from low-quality peer review or lack peer review altogether, citations to these papers can propagate errors or pseudoscience into the literature. Inspections reveal that papers in predatory journals have infiltrated citation databases, such as PubMed and Scopus, with some predatory journals being indexed in these databases, potentially garnering citations from legitimate journals.^15^

 Scopus removes journals from its list due to metrics (i.e., self-citation rate), publication concerns (Concerns or comments regarding the quality of publications), or radar (an algorithm that identifies the outlier performance of a journal).^16,17^ Various investigations identified publications excluded from Scopus for “Publication Concerns” as potential predatory journals or journals exhibiting predatory behaviours.^17-19^ In other words, discontinued titles from Scopus are not automatically considered predatory journals, but many journals discontinued for ‘publication concerns’ or similar quality issues exhibit characteristics associated with predatory practices.^17-19^ Most of the discontinued journals by Elsevier are due to “publication concerns” rather than “metrics,” and it is assumed that many of these journals are predatory or have similar practices.^20^

 Citations to papers published in discontinued titles can be detrimental, as the content from these journals may be disseminated through other publications.^21^ This issue is more critical when the reason for discontinuation is unethical publishing practices or predatory behaviour.^20,21^

## The Gap in the Literature

 Despite numerous studies examining the influence of predatory journals and discontinued titles from Scopus, as well as the dissemination of their published papers within academic literature, there is a lack of information about the impact of papers published in these journals outside academia. Most of the research focuses on paper-to-paper citations, primarily by considering published papers in predatory journals or discontinued titles from Scopus as the cited papers, and attempts to identify citation patterns and raise awareness to prevent future citations of non-peer-reviewed or poorly reviewed science.^15,18,19,22-24^

 Patents will also cite academic papers. Patent-to-paper citations serve as a proxy indicating the transfer of knowledge from academia to industry.^25^ The patent analysis helps to understand future trends in the industry and identify a path to technological progress.^25,26^ Research has examined patent-to-paper citations to assess the technological influence of literature.^9,27,28^ The inclusion of citations from predatory/discontinued titles (potentially predatory journals) in patent literature raises substantial concerns about the credibility of the scientific foundation for claimed ideas. While much of the existing academic discourse focuses on the issue of citing questionable journal papers in non-patent literature (such as academic papers, conference proceedings, books, and book chapters), there is a notable gap in research that quantifies the extent of such citations within patents and assesses the resulting harm.

## Methods

 For this study, the list of discontinued titles from Scopus was used to identify potential predatory medical journals. In current editorial, we considered journals from the Scopus list of terminated titles that were removed due to publication concerns. The publication types are limited to journals (not books, trade journals, etc.). We exclusively considered journals with ‘Medicine’ as their primary ‘Subject Area and Category’ according to *SCImago* (https://www.scimagojr.com). Furthermore, our selection was limited to journals that were delisted from the index in 2020 or later. The data on discontinued titles were collected from Scopus (https://downloads.ctfassets.net/o78em1y1w4i4/7xtaTxNiNcWRTeZkV86eNy/94df1090585ba656419b5af2b1440b21/ext_list_May_2025.xlsx). We then used available lists of potential predatory journals to verify which of the discontinued journals had been flagged as predatory. Sources included websites such as https://www.predatoryjournals.org and https://beallslist.net. The Lens database (https://www.lens.org) was used to analyse the citations garnered by these potential predatory journals. The Lens offers free access to scholarly publications and patents, featuring distinctive functions that facilitate a thorough examination of citations between papers and patents.^29-31^

## Results

 Applying a filter for journals delisted in 2020 or later due to publication concerns yielded 200 discontinued titles. When this list was further refined by the primary ‘Subject Area and Category’ of ‘Medicine,’ 26 journals were identified. Finally, after considering the available block lists, eight journals were identified as potential predatory medical journals including *Applied Bionics and Biomechanics, Clinical Schizophrenia and Related Psychoses, Computational Intelligence and Neuroscience, Current Pediatric Research, Disease Markers, Evidence-based Complementary and Alternative Medicine, International Journal of Biology and Biomedical Engineering*, and *Journal of Environmental and Public Health*. To discover relevant publications, Lens looked up both the print ISSN (International Standard Serial Number) and the electronic ISSN of these eight journals (The search date was 1 August 2025). The result provided 29,609 papers that received 483,848 citations from scholarly works and 4,251 citations from patents.

**Figure 1:**
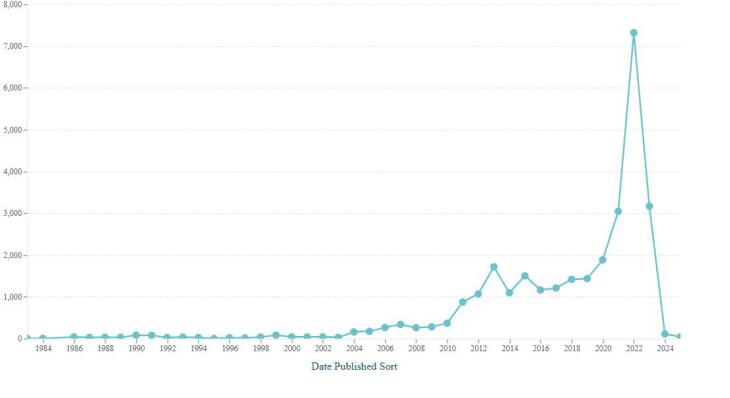
The total number of published papers per year by the potential predatory medical journals

Figure 1 illustrates the annual publication output of the potential predatory medical journals. A notable decrease in total publications is observed in 2024, likely reflecting authors’ awareness of these journals’ delisting from Scopus.

**Figure 2:**
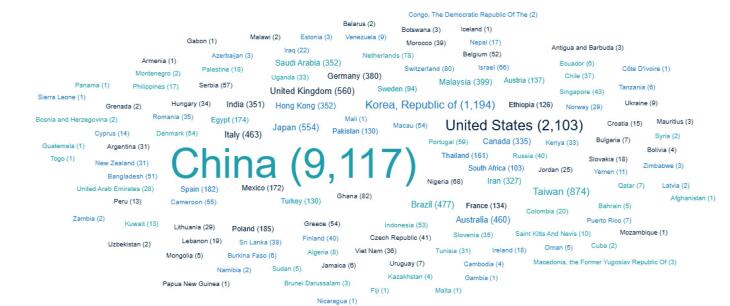
Most active countries in publishing in potentially predatory medical journals

 Ninety-nine percent of the publication papers are journal articles, and the others are editorial, letters, news, etc. A considerable number of publishing authors come from China; however, the number of authors from the USA, the Republic of Korea, and the UK exceeds that from other countries (Figure 2).

**Figure 3:**
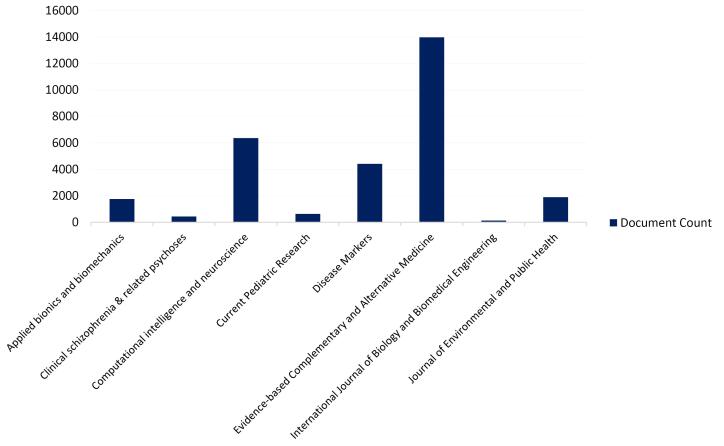
Potential predatory medical journals based on the number of published papers

Figure 3 illustrates journals based on the number of papers they have published. *The Evidence-Based Complementary and Alternative Medicine* is one of the most active journals, having published over 13,000 papers.

**Figure 4:**
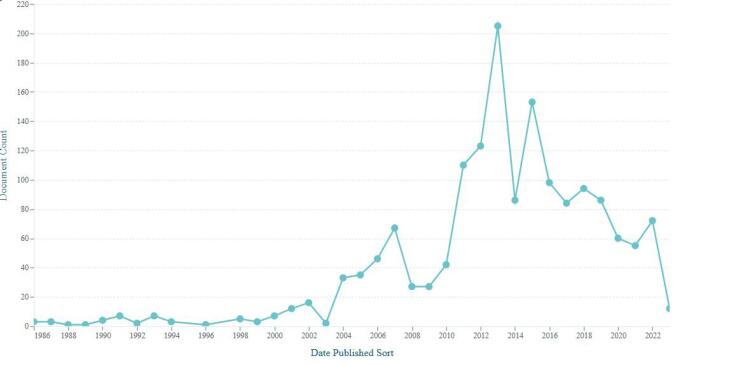
The distribution of publication years for scholarly works cited by patents

 The 1,592 scholarly works in the dataset have been cited by 4,009 unique patents, which collectively received 4,251 patent citations. Most of these cited scholarly works were published between 2011 and 2022 (Figure 4). Most of the authors of these cited scholarly works are from China (406), the USA (204), Taiwan (125), the Republic of Korea (109), and other countries. Three journals accounted for more than 93 percent of cited papers by patents, including *Evidence-based complementary and alternative medicine* (843 papers), *Disease markers* (385 papers), and *Computational intelligence and neuroscience* (268 papers). The 20 scholarly works cited by patents (with a total of 26 patent citations) have been retracted. There are also 12 patent citations for papers that were published after 2023 in these potential predatory medical journals.

**Figure 5:**
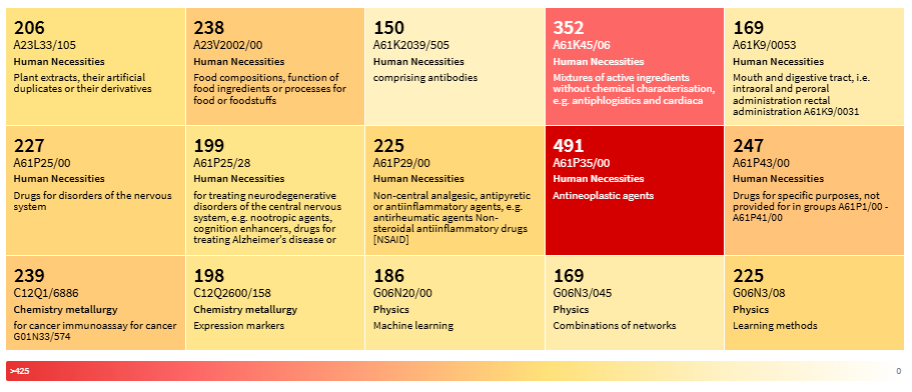
The top frequent CPC codes found in citing patents

 From the 4,009 unique patents citing papers in the dataset, data for 3,962 patents were successfully retrieved from Lens for subsequent analysis. Figure 5 depicts the most common Cooperative Patent Classification (CPC) codes found in cited patents. To facilitate patent examination, searching, and study worldwide, the European Patent Office (EPO) and the United States Patent and Trademark Office (USPTO) partnered to create CPC codes, which are classified themes based on technology.^32^ The heatmap (Figure 5) highlights the primary technological domains impacted by a specific set of discoveries, displaying the most commonly used CPC codes in citing patents. The significant influence on healthcare and drug development is highlighted by dominant high-frequency codes, which are primarily found in “Human Necessities” such as medications and medical treatments (e.g., A61K45/06 for active ingredient mixes, A61P35/00 for antineoplastic drugs). The presence of codes from “Chemistry Metallurgy” (e.g., C12Q1/6886 for cancer immunoassay) and “Physics” (e.g., G06N20/00 for machine learning) suggests a broader, multidisciplinary importance, even though the medical sciences remain essential. The technological environment and the varied impact of the patents are succinctly summarised in this graphic.

## Discussion

 The findings of the present study offer novel insights into aspects that have been previously overlooked in the literature. Current editorial has identified eight potential predatory medical journals that could attract considerable citations from the patent literature. It means that potential predatory journals can influence the transfer of science from academia to industry and industrial innovation. This study raises a significant alarm about technological innovation, particularly within the medical domain, where technological advancements have a direct impact on human health. A critical question thus emerges: *Is it time for organizations like the EPO or the USPTO to implement measures against the citation of potential predatory journals?* The inclusion of non-peer-reviewed or pseudo-scientific literature in patents poses a significant threat to technological progress, particularly since published patents and scientific literature are often used to predict future innovative technologies. Specifically, the citation of papers from potential predatory journals by patents can disseminate flawed information. Such unreliable ‘prior art’ could lead to the erroneous rejection of legitimate patent applications and, more broadly, result in a substantial waste of resources—both financial and temporal—for inventors and investors alike.

 Another interesting result from the current study is the geographical distribution of authors who have previously published in potential predatory journals. Our sample data indicate that most authors originate from China, the USA, Indonesia, and the Republic of Korea. This observation diverges from the prevailing research, which primarily suggests that most authors exploited by predatory journals originate from low-income countries.^17,33-35^ The prior indexing of suspected predatory journals may impact the geographical distribution of authors who have been victimised. However, the larger dataset of potential predatory journals should be used to provide more generalised conclusions regarding victim authors of previously indexed potential predatory journals. It can be a topic for future research. The indexation of a journal within a reputable scientific database, such as Scopus, typically serves as a strong indicator of its credibility for authors. Consequently, predatory journals that successfully achieve Scopus indexation are likely to attract a higher proportion of authors from developed countries. Some of these journals initially present a legitimate facade and actively seek to be indexed. However, once indexed, they often transition into high-volume ‘publishing machines,’ prioritizing the monetization of article processing charges. Fortunately, the continuous evaluation process employed by Scopus eventually identifies and delists such journals.

## Conclusion

 The findings suggest that patents can cite papers published in potential predatory medical journals, thereby amplifying the adverse implications of these journals beyond the academic sphere. Patent literature is crucial for fostering technological innovation, forecasting future technologies, and protecting intellectual property rights. When patents cite papers from potential predatory medical journals, it risks spreading flawed information. This may lead to erroneous rejections of legitimate patent applications, ultimately resulting in a waste of resources—both financial and temporal—for inventors and investors.

 The current study has several limitations. First, it only included journals delisted from Scopus due to publication concerns and verified their potential predatory status using available lists. This approach reflects a strict method of identifying potential predatory medical journals by cross-referencing two sources. While broader sources—such as the archived Beall’s List or the Scopus discontinued list alone—may offer more comprehensive coverage and introduce some variation in specific data points, the core finding remains consistent: patents cite papers published in potential predatory journals. Second, we did not investigate when a particular journal began engaging in predatory practices, whether from its inception or only in recent years. However, Figure 4, which shows the publication years of papers cited by patents, may offer insight into how recently published papers in potential predatory medical journals have been cited. Finally, we did not examine how the cited papers were used in the patents, whether as foundational references or simply as background information. However, such limitations are common in studies analyzing citations to predatory journals in the literature.

 Future research could explore the risks associated with patents citing papers published in hijacked journals, where cybercriminals establish counterfeit websites for legitimate publications.^36-38^

## Competing Interests

 None declared.

## Ethical Approval

 Not applicable.
